# Rhinitis, sinusitis and ocular disease – 2098. Exhaled NO may predict development of allergic rhinitis in children with asthma

**DOI:** 10.1186/1939-4551-6-S1-P174

**Published:** 2013-04-23

**Authors:** Yeong Ho Rha, Han Seok Ko

**Affiliations:** 1 Department of Pediatrics, Kyung Hee University Hospital, Seoul, South Korea

## Background

As a non-invasive parameter of lower airway inflammation, fraction of exaled nitric oxide (FeNO) concentration has been known to be related with bronchial hyperreactivity in asthma patient. FeNO may be increased in atopy related diseases (e.g. allergic rhinitis) but relationship of FeNO and development of allergic rhinitis in asthma is unknown. The aim of this study was to investigate whether measurement of FeNO in asthma children can predict development of allergic rhinitis.

## Methods

Fifty-three children with mild to moderate persistent asthma aged from 5 to 15 years who were measured with FeNO, total eosinophil count and IgE were included. FeNO was measured through chemiluminescence analyzer. Prospectively, the patients were followed after 6 years by interview with questionnaire and FeNO levels of the patient who developed allergic rhinitis (allergic rhinitis group and who do not manifest allergic rhinitis (control group) were evaluated.

## Results

There were no difference of peripheral blood total eosinophil count, serum IgE, age, sex, family history, history of atopic dermatitis, or degree of asthma severity between allergic rhinitis group and control group. FeNO was significantly higher in allergic rhinitis group compared to control group (29.4 vs 24.6 parts per billion [ppb] vs 13.6 vs 11.8 ppb; p = 0.003).

## Conclusions

Measurement of FeNO can be a useful tool to predict to predict development of allergic rhinitis in asthmatic children.

